# A bioinformatics investigation into the pharmacological mechanisms of javanica oil emulsion injection in non-small cell lung cancer based on network pharmacology methodologies

**DOI:** 10.1186/s12906-020-02939-y

**Published:** 2020-06-05

**Authors:** Mengwei Ni, Xinkui Liu, Ziqi Meng, Shuyu Liu, Shanshan Jia, Yingying Liu, Wei Zhou, Jiarui Wu, Jingyuan Zhang, Siyu Guo, Jialin Li, Haojia Wang, Xiaomeng Zhang

**Affiliations:** Department of Clinical Chinese Pharmacy, School of Chinese Materia Medica, Beijing University of Chinese Medicine, No. 11 of North Three-ring East Road, Chao Yang District, Beijing, China

**Keywords:** Traditional Chinese medicine, Javanica oil emulsion injection, Non-small cell lung cancer, Network pharmacology, Molecular docking

## Abstract

**Background:**

Javanica oil emulsion injection (JOEI) is an effective therapeutic option for patients with non-small cell lung cancer (NSCLC), but its mechanisms have not been fully elucidated.

**Methods:**

In this study, we utilized network pharmacology to systematically investigate the bioactive components and targets of JOEI, identify common targets in NSCLC, and understand and evaluate the underlying mechanism of JOEI in the treatment of NSCLC through expression level, correlation, enrichment, Cox, survival and molecular docking analyses. The results indicated that five compounds of JOEI interact with five pivotal targets (LDLR, FABP4, ABCB1, PTGS2, and SDC4) that might be strongly correlated with the JOEI-mediated treatment of NSCLC.

**Results:**

The expression level analysis demonstrated that NSCLC tissues exhibit low expression of FABP4, ABCB1, LDLR and PTGS2 and high SDC4 expression. According to the correlation analysis, a decrease in FABP4 expression was strongly correlated with decreases in LDLR and ABCB1, and a decrease in LDLR was strongly correlated with decreased PTGS2 and increased in SDC4 expression. Cox and survival analyses showed that the survival rate of the high-risk group was significantly lower than that of the low-risk group (*p* = 0.00388). In the survival analysis, the area under the curve (AUC) showed that the pivotal gene model exhibited the best predictive capacity over 4 years (AUC = 0.613). Moreover, the molecular docking analysis indicated that LDLR, FABP4, ABCB1, PTGS2 and SDC4 exhibit good binding activity with the corresponding compounds.

**Conclusion:**

In conclusion, this study predicted and verified that the mechanism of JOEI against NSCLC involves multiple targets and signaling pathways. Furthermore, this study provides candidate targets for the treatment of NSCLC, lays a good foundation for further experimental research and promotes the reasonable application of JOEI in clinical treatment.

## Background

Based on global estimates, 18.1 million new cancer cases and 9.6 million deaths occurred in 2018. As one of the most common cancers globally, lung cancer remains a critical cause of cancer-related death [1, 2]. Among smokers and nonsmokers, non-small cell lung cancer (NSCLC) is the most common subtype of lung cancer [3]. Due to the limitation of its early detection, the majority of patients with NSCLC are diagnosed at late stages, and the 5-year overall survival (OS) rate is only 11% [4–6].. Therefore, an in-depth understanding of the regulatory mechanism of NSCLC occurrence and development is urgently needed to provide more effective strategies for the treatment of this type of cancer.

Traditional Chinese medicine (TCM), as a crucial component of complementary and alternative medical systems, has been widely applied in Asian nations, particularly China, Japan and North and South Korea, for thousands of years for the clinical treatment of cancers [7, 8]. In particular, herbal medicine is considered part of the anticancer strategy in China. A large number of cancer patients prefer to receive TCM via either injection or oral administration when receiving radiotherapy or chemotherapy, [9, 10].

With the continuous expansion of clinical practice toward the comprehensive treatment of tumors, TCM has been proven to be effective in not only relieving adverse events such as fatigue, pain, emesis, diarrhea, and pancytopenia caused by surgery and chemotherapy but also improving quality of life and immune functions and strengthening survival benefits [8, 11–14].

Javanica oil emulsion injection (JOEI), which is a product produced from Brucea oleifera ether extracts as a raw material, has been engaged as an adjunctive therapy for lung carcinoma, brain metastasis of lung carcinoma, and gastrointestinal tumorigenesis in China [15–19]. Recent studies have recognized that some components of JOEI exhibit specific affinity for tumor cell membranes and potent antitumour activity [20]. Previous investigations have indicated that JOEI can enhance efficacy, improve quality of life and decrease the incidence of platinum-containing chemotherapeutic side effects, such as nausea, vomiting and leukopenia, for patients with advanced NSCLC in the clinic, but no relevant study has attempted to explain its mechanism [16, 21]. To enhance the treatment effect of JOEI, the molecular and biological basis of JOEI in the treatment of NSCLC needs to be elucidated [22].

Network pharmacology has recently emerged as a novel strategy for identifying the bioactive compounds of several TCM formulas and their underlying complex pharmacological mechanisms from systemic and holistic perspectives [12, 23–27]. Network pharmacology has been applied to delineate the convoluted interactions among genes, proteins and metabolites related to diseases and drugs from the perspective of networks, which is in line with the multicomponent and multitarget nature of TCM. The integration of network pharmacology and TCM alters the conventional “one target, one drug” paradigm to a “multi-target, multi-component drug” strategy [28].

**Fig. 1:**
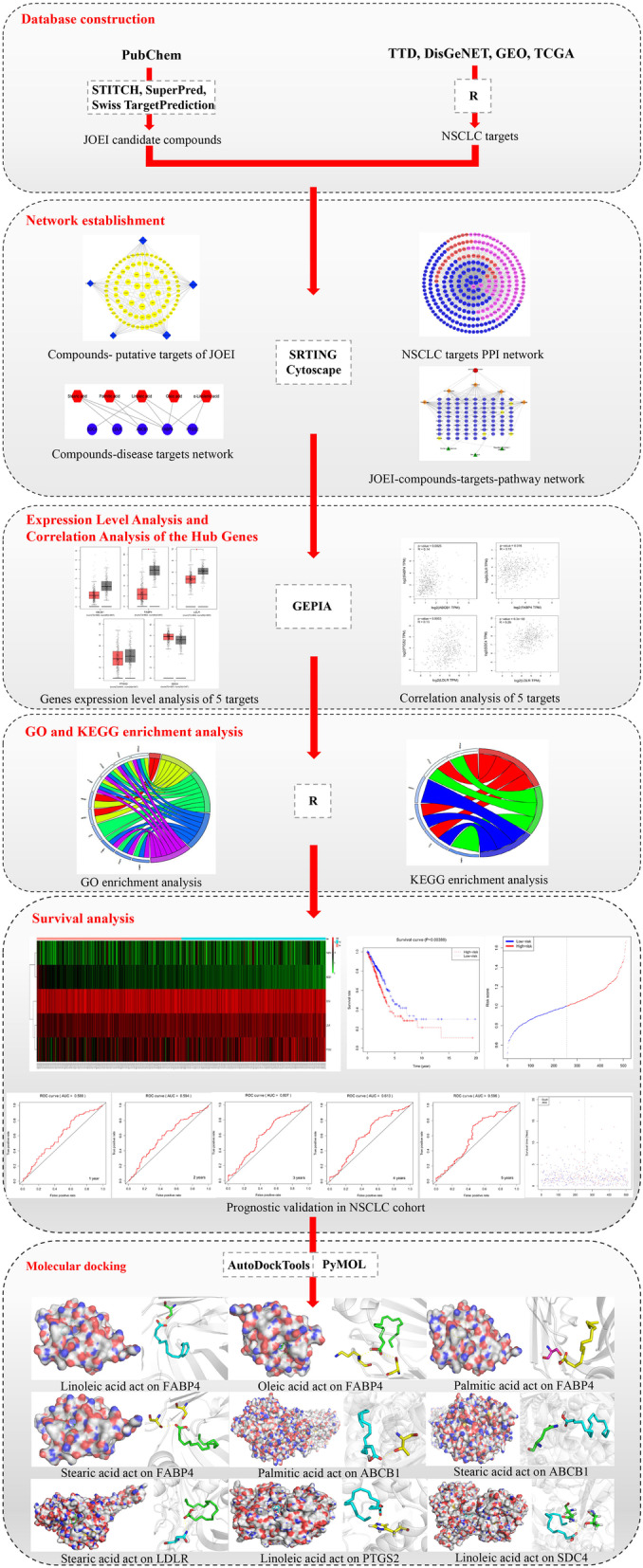
Flowchart of the technical strategy

Therefore, in this study, we employed network pharmacology and bioinformatics methods to investigate and predict the molecular mechanisms underlying the effectiveness of JOEI against NSCLC. A flowchart of the technical strategy used in this study is presented in Fig. 1.

## Methods

### Active components and putative targets of JOEI

By a thorough literature review, we aimed to identify and extract the chemical composition of JOEI [8, 10, 11]. All the compounds were then inputted into the PubChem database (https://pubchem.ncbi.nlm.nih.gov) [29] to obtain their respective 3D molecular structure files. Because the targets of the compounds without accurate structural information could not be successfully predicted, we decided to exclude these chemicals after removing replicated data. The 3D chemical structure files of all the active compounds were imported into the Search Tool for Interacting Chemicals (STITCH, http://stitch.embl.de/) [30, 31], SuperPred (http://prediction.charite.de/) [32], and SwissTargetPrediction (http://www.swisstargetprediction.ch/) [33]. To obtain the corresponding known or predicted targets from the above-mentioned three databases after discarding duplicated data, only human targets were analyzed.

### Known targets related to NSCLC

The human targets affiliated with NSCLC can be obtained from four resources:

(1) The Therapeutic Target Database (TTD, https://db.idrblab.org/ttd/) is a database that furnishes information on acknowledged and explored therapeutic proteins and targeted diseases, nucleic acid targets and pathways as well as the corresponding drugs directed at each of these targets [34]. We screened the TTD using the keyword “non-small cell lung cancer” and acquired 54 known NSCLC-related targets.

(2) DisGeNET is a discovery platform that integrates information from various data sources. DisGeNET provides information on diseases, gene-disease associations, clinical or abnormal human phenotypes, disorders, variant-disease associations, and traits, among other data, to support studies on the mechanisms underlying human diseases [35].

(3) The Gene Expression Omnibus (GEO, http://www.ncbi.nlm.nih.gov/geo/) is an international overt repository that archives and freely distributes high-throughput gene expression and other functional genomics datasets [36]. Datasets that met the following criteria were potentially included: 1) tissue samples collected from human NSCLC and corresponding adjacent or normal tissues and 2) 30 samples at any rate. For assaying decontrolled gene expression, the differentially expressed genes (DEGs) were identified using the “limma” package of R software [37], and the DEGs in each microarray were also filtered using the same package. Target integration of the DEGs discriminated from four datasets (GSE19804, GSE18842, GSE43458, and GSE62113) was performed using RobustRankAggreg [38]. Genes with a log2-fold change |log_2_FC| ≥1 and an FDR-adjusted *P* value < 0.05 were considered DEGs [39].

(4) The Cancer Genome Atlas (TCGA) provides over 2.5 petabytes of genomic, epigenomic, transcriptomic, and proteomic data, and our ability to diagnose, treat, and prevent cancer has benefited from these data [40]. We obtained the NSCLC-related database from https://xenabrowser.net/datapages/, clicked on “gene expression RNAseq”, selected “HTSeq - Counts (n=585) GDC Hub”, clicked on “phenotype” and chose “Phenotype (*n*=877) GDC Hub”, and the resulting database was analyzed using the ‘edgeR’ package in R [41].

### Protein-protein interaction (PPI) analysis

We inputted the NSCLC-related targets and putative targets of certain chemical components into the Search Tool for the Retrieval of Interacting Genes/Proteins (STRING, https://string-db.org/) database [42], which is a database of known and predicted PPIs that includes both direct and indirect interactions among proteins. After restricting the species to “*Homo sapiens*”, PPI data with confidence scores above 0.7 (low: < 0.4; medium: 0.4 to 0.7; and high: > 0.7) were identified as putative targets for further research.

### Network construction

The network visualization tool Cytoscape 3.6.1 (http://cytoscape.org/, ver. 3.5.1) was adopted to obtain the PPI network map [43]. Common targets between the compound-putative target network and the NSCLC target PPI network were identified as potential targets for the components of JOEI in NSCLC. In such a network, an injection, a compound, or a gene/protein serves as an “edge” and reflects an association between nodes. For each node in the interaction network, three indices (significant parameters) were measured to assess its topological features: degree, betweenness, and closeness. The measure “degree” is construed as the number of edges associated with node i, and nodes with a higher degree are considered more important. The metric “betweenness” represents the number of shortest paths between node pairs passing through node i, and the measure “closeness” is the reciprocal of the sum of the distances from node i to other nodes. Nodes with higher values of these measures are more important in the network. The key hubs in the network all exhibit high centrality [44–47].

### Expression level and correlation analyses of the key targets

The results from the expression level and correlation analyses were visualized through Gene Expression Profiling Interactive Analysis (GEPIA) (http://gepia.cancer-pku.cn/index.html), a web-based tool that analyzes tumor data from TCGA and the Genotype-Tissue Expression (GTEx) project. GEPIA contains 9736 tumor samples and 8587 normal tissue samples covering 33 malignancies. The expression analysis between tumor and normal data was performed using a standard processing pipeline [48], which allows both an analysis according to specific conditions, similar to tumor and normal differential expression analysis, and the detection of the expression of the hub targets in NSCLC and normal tissues. A boxplot was then generated to visualize the relationships [49].

### Enrichment analysis

To clarify the roles of the potential targets in gene function and signaling pathways, Gene Ontology (GO) enrichment and Kyoto Encyclopedia of Genes and Genomes (KEGG) pathway enrichment analyses of the targets in the compound-NSCLC target network were performed using the g:Profiler (https://biit.cs.ut.ee/gprofiler/gost) database. The GO project divided functions into three facets, cellular component, molecular function and biological process, and reveals possible biological processes associated with key targets [50, 51]. In addition to pathway enrichment analysis, KEGG pathway enrichment analysis provides pathway functional annotations of a specific gene set. According to the results from the g:Profiler database, the multi-component, multi-target and multi-pathway characteristics of JOEI for treating NSCLC can be illustrated by analyzing the vital GO terms and pathways of the key targets. The results from the GO and KEGG analyses were visualized using the ‘GOplot’ package in R software [52].

### Cox and survival analyses

The risk score (RS) was based on the linear combination of the candidate mRNAs for each patient with NSCLC and calculated by multiplying the sum of the mRNA expression values by the single variable Cox regression coefficient [39].

The prediction performance of the model was measured based on the area under the curve (AUC) obtained from the time-dependent receiver operating characteristic (ROC) analysis, and the accuracy of the RS to predict OS at 1 to 5 years was assessed. The capacity of the evaluation model can be obtained by analyzing the AUC of the ROC curve. A larger AUC usually represents better performance, and the AUC was greater than 0.7, which indicated that the model has better classification capacity. All statistical analyses were performed using R software (version 3.4.2), and survival and ROC curves were drawn using the ‘survival’ and ‘survivalROC’ packages, respectively [53, 54].

### Molecular docking

Molecular docking has been extensively used for ligand-based and structured-based target prediction. The 3D crystal structures of the candidate targets from the Research Collaboratory for Structural Bioinformatics (RCSB) Protein Data Bank (http://www.pdb.org/) [55] were downloaded to assess these targets. If the root mean square deviation (RMSD) of a model is less than 3 Å, it can be regarded as a decent or precise model (accurate ≤2 Å, reliable ≤4 Å) [56]. The protein structures were prepared using AutoDockTools (ADT) [57], and this analysis includes removing ligand and water molecules, computing Gasteiger charges, adding polar hydrogens, and merging nonpolar hydrogens. The results were then saved in MOL2 format. The compounds were also prepared using ADT, and a Gasteiger charge was assigned to the compounds. The prepared protein structures and compounds were saved in PDBQT format. Molecular docking was then performed with AutoDock Vina, and the results were viewed and analyzed using PyMOL (http://www.pymol.org) [58].

## Results

### Compound-putative target network

**Fig. 2:**
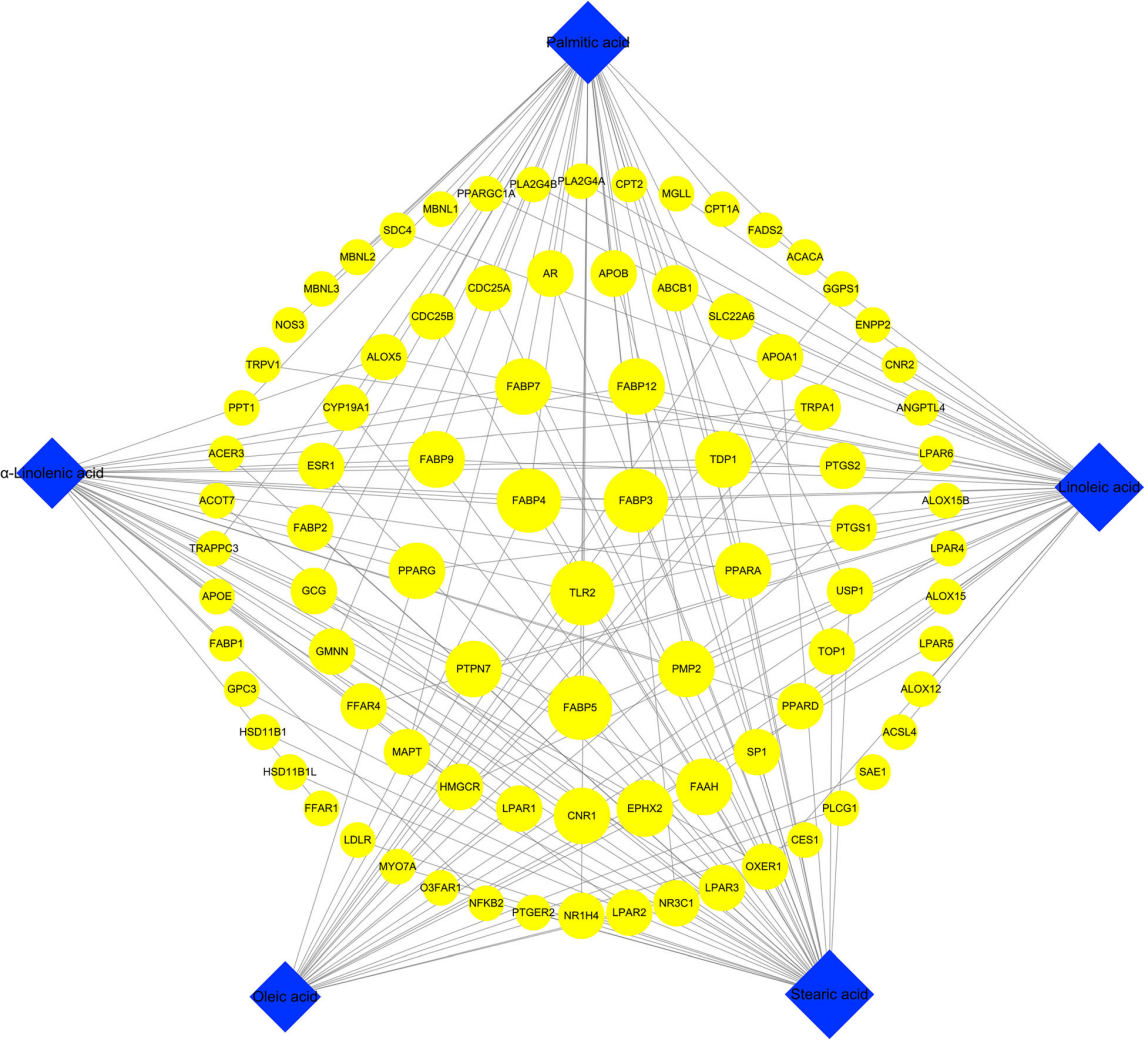
Compound-putative target network of JOEI Note: The blue diamonds represent compounds contained in JOEI. The yellow ellipses represent the corresponding targets. The node size has a direct proportional (positive) relationship with the degree.

Large-scale text mining identified a total of seven active components in JOEI. After the initial analysis, five active compounds with structural information and 87 putative targets were selected for further study. Detailed information of the five compounds in JOEI is described in Table 1. The compound-putative target network (shown in Fig. 2) was constructed with 92 nodes (five compound nodes and 87 putative target nodes) and 160 edges. In this network, many putative targets were associated with multiple compounds (to trigger their biological effects and vice versa); therefore, the targets that play a pivotal role in the whole network might be considered key compounds or targets. The network pharmacology analysis of all the compounds revealed that the compounds with the highest degrees were linoleic acid and palmitic acid, followed by stearic acid, α-linolenic acid and oleic acid.

**Table 1:** Information on the chemical components of JOEI

PubChem CID	COMPOUND	Canonical SMILES	STRUCTURE
5280450	Linoleic acid	CCCCCC=CCC=CCCCCCCCC(=O)O	
985	Palmitic acid	CCCCCCCCCCCCCCCC(=O)O	
5281	Stearic acid	CCCCCCCCCCCCCCCCCC(=O)O	
445639	Oleic acid	CCCCCCCCC=CCCCCCCCC(=O)O	
5280934	α-Linolenic acid	CCC=CCC=CCC=CCCCCCCCC(=O)O

### PPI network of NSCLC targets

**Fig. 3:**
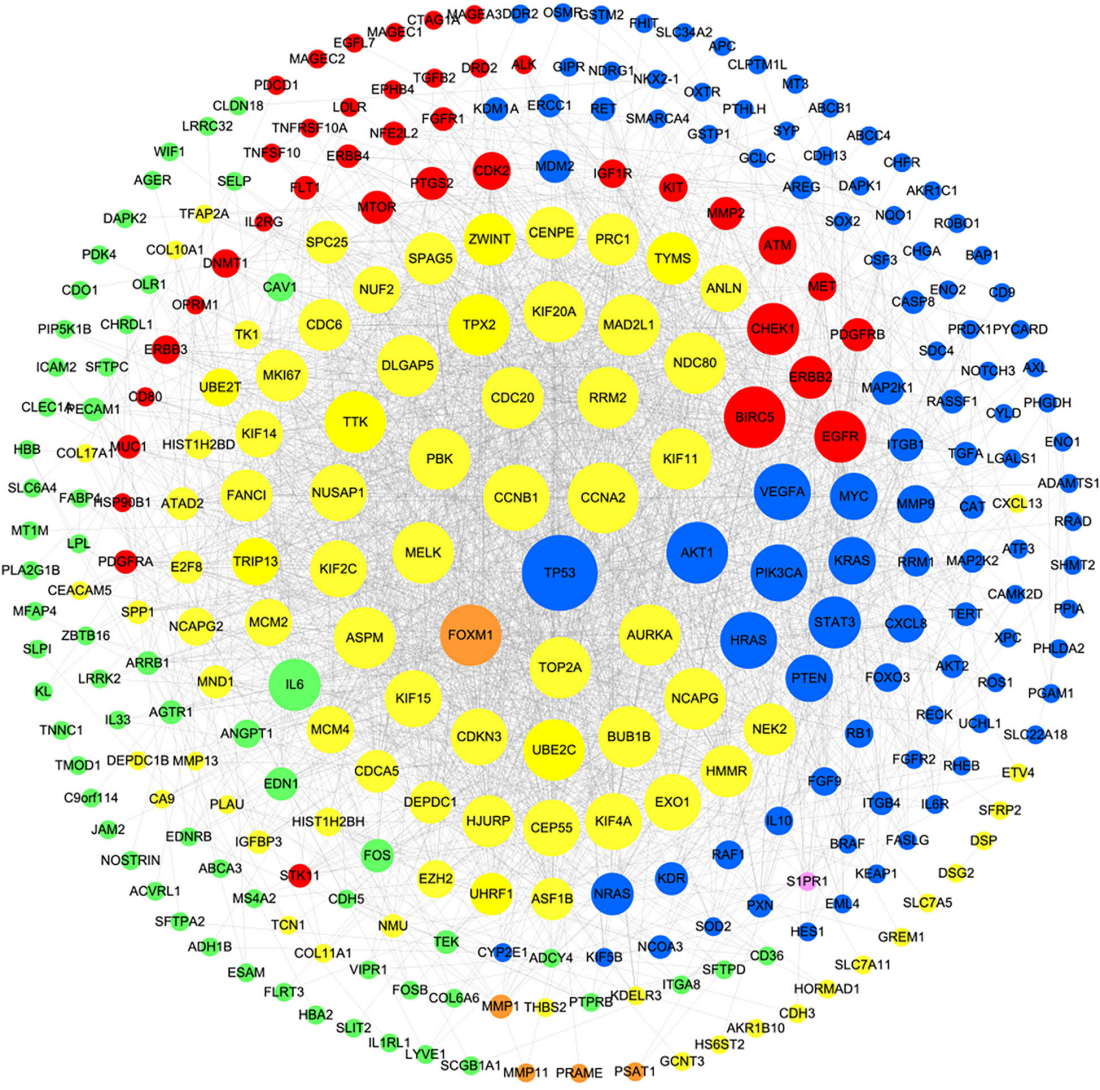
PPI network related to NSCLC. Note: The red ellipses represent targets obtained from the TTD. The pink ellipses represent targets obtained from TCGA-down, GEO and TTD. The blue ellipses represent targets obtained from the TTD and DisGeNET. The green ellipses represent targets obtained from TCGA-down and GEO. The yellow ellipses represent targets obtained from TCGA-up and GEO. The orange ellipses represent targets obtained from TCGA-up, TTD, DisGeNET and GEO. The node size has a direct proportional (positive) relationship with the degree

Note: The red ellipses represent targets obtained from the TTD. The pink ellipses represent targets obtained from TCGA-down, GEO and TTD. The blue ellipses represent targets obtained from the TTD and DisGeNET. The green ellipses represent targets obtained from TCGA-down and GEO. The yellow ellipses represent targets obtained from TCGA-up and GEO. The orange ellipses represent targets obtained from TCGA-up, TTD, DisGeNET and GEO. The node size has a direct proportional (positive) relationship with the degree.

A total of 406 NSCLC targets were retrieved from the TTD, DisGeNET, TCGA and GEO databases. As shown in Fig. 3, the PPI network of NSCLC targets depicts the interactions between the 292 target proteins ultimately identified from the STRING database. The major nodes were identified by calculating three topological features for each node in the network.

### Identification of core genes and network analysis

**Fig. 4:**
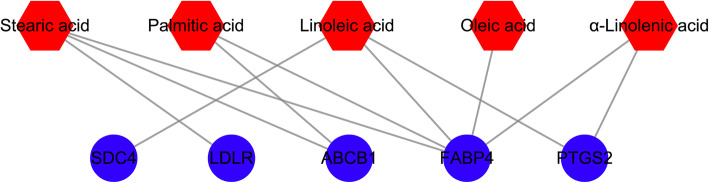
Compound-NSCLC target network. Note: The red hexagons represent compounds of JOEI, and the blue circles represent potential targets of JOEI against NSCLC

To further unveil the therapeutic mechanism of JOEI resistance in NSCLC, the overlapping genes between compound and NSCLC targets were identified. We found five genes in both the list of NSCLC targets and the list of putative targets (Table 2). As displayed in Fig. 4, the compound-NSCLC target network involved 10 nodes (five common targets and five corresponding chemical components) and 11 edges, which indicated that the 10 nodes might act as potential targets for the treatment of NSCLC with JOEI.

**Table 2:** Compound-NSCLC target network

UniProt accession	Gene name	Protein name	Structure
P01130	LDLR	Low-density lipoprotein receptor	
P08183	ABCB1	Multidrug resistance protein 1	
P31431	SDC4	Syndecan-4	
P35354	PTGS2	Prostaglandin G/H synthase 2	
P15090	FABP4	Fatty acid-binding protein, adipocyte

### Expression level and correlation analyses of the key targets

The prognostic information for the five key genes is available for free in the GEPIA database.

**Fig. 5:**
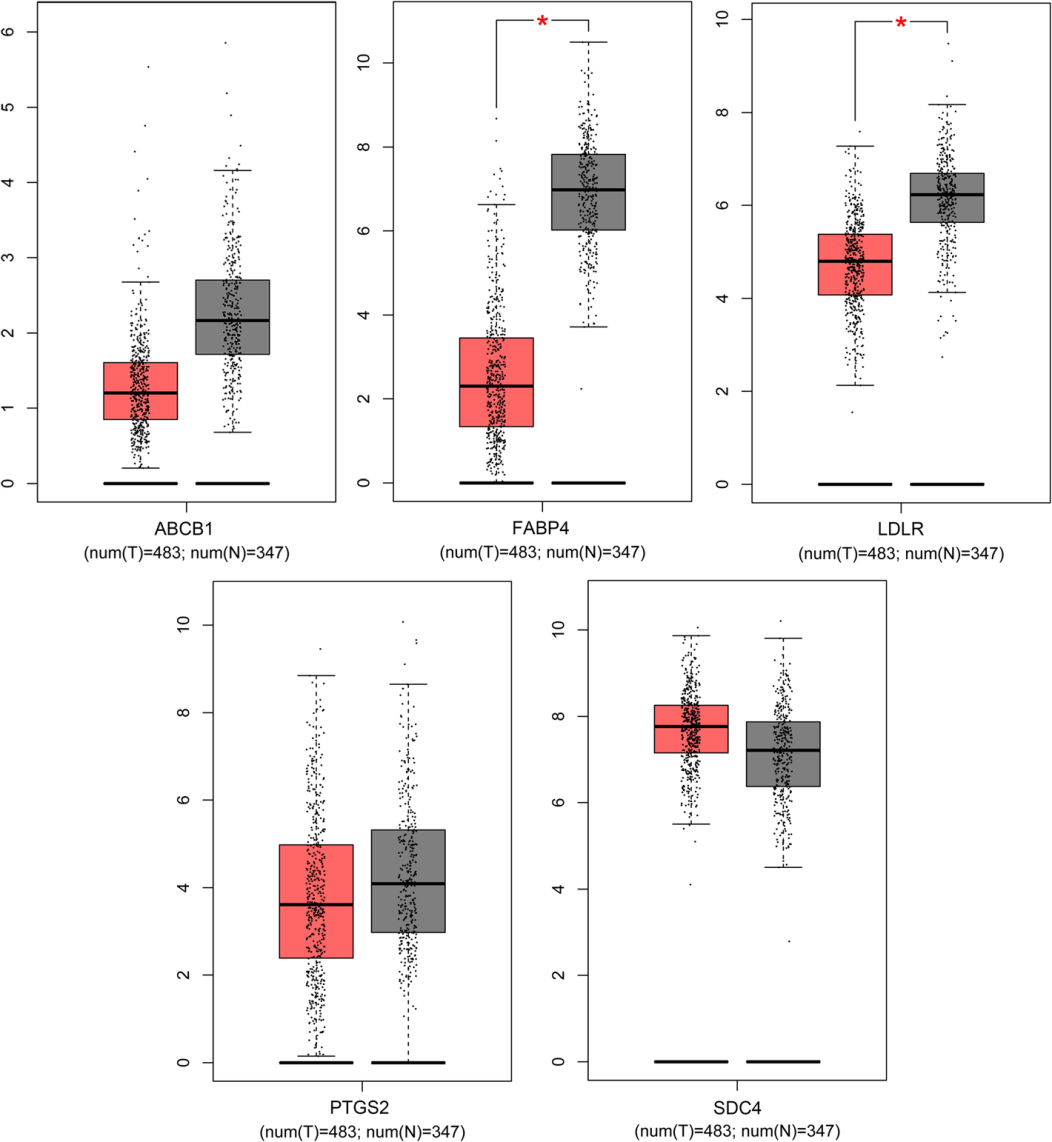
Gene expression level analysis of 5 hub genes in human NSCLC. Note: Red and gray boxes represent normal and cancer tissues, respectively

We subsequently employed GEPIA to examine the differences in hub gene expression between NSCLC and normal tissues, as shown in Fig. 5.

**Fig. 6:**
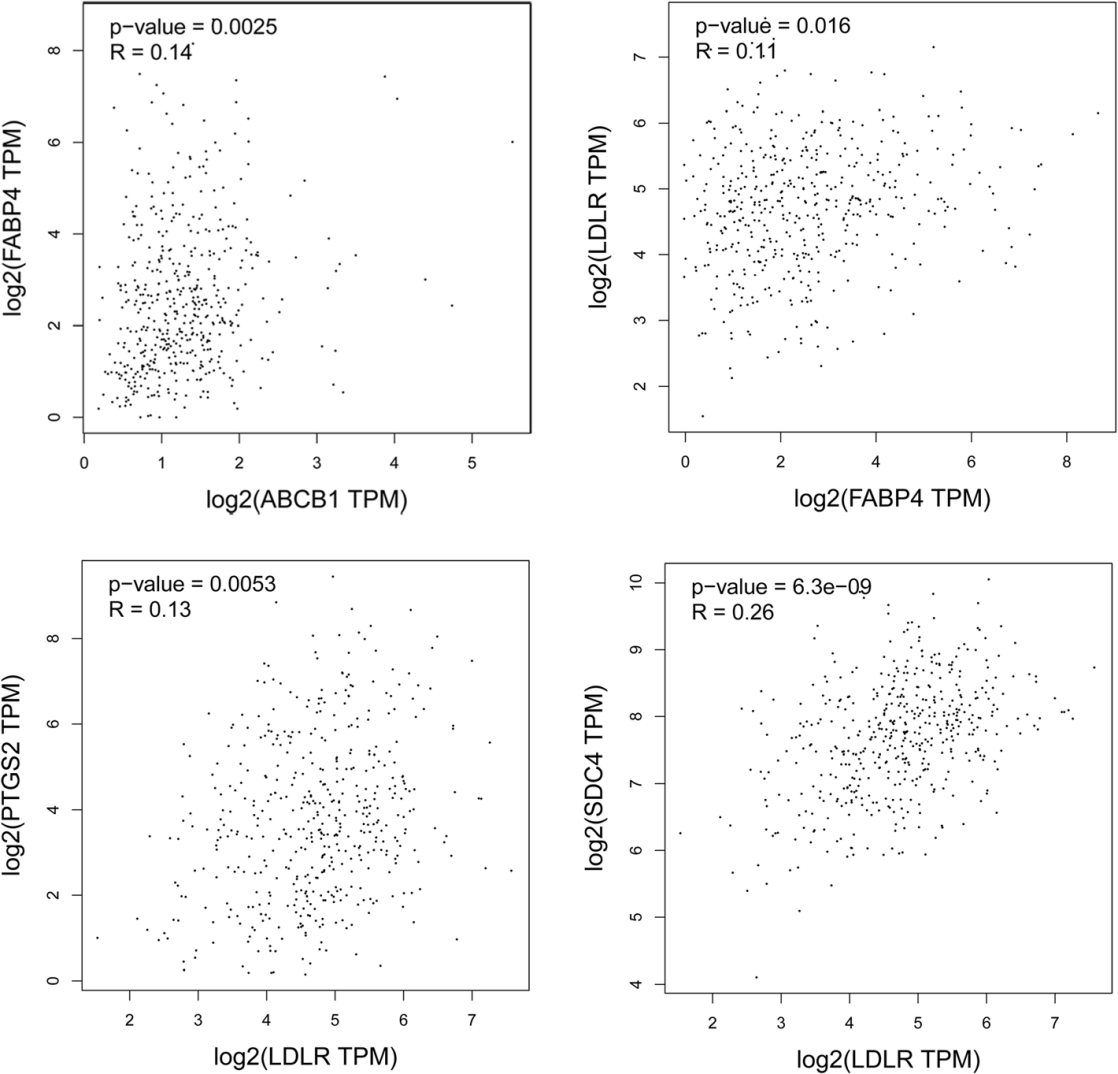
Correlation analysis of five hub genes in human NSCLC

Fatty acid-binding protein 4 (FABP4), ATP-binding cassette subfamily B member 1 (ABCB1), low-density lipoprotein receptor (LDLR) and prostaglandin endoperoxide synthase 2 (PTGS2/COX-2) are highly expressed in NSCLC tissues, and the expression of syndecan 4 (SCD4) in these tissues was low (Fig. 5). A decrease in FABP4 expression was strongly correlated with decreases in LDLR and ABCB1, and a decrease in LDLR was strongly correlated with decreased PTGS2 and increased in SDC4 expression (Fig. 6).

### GO and KEGG pathway enrichment analyses

**Fig. 7:**
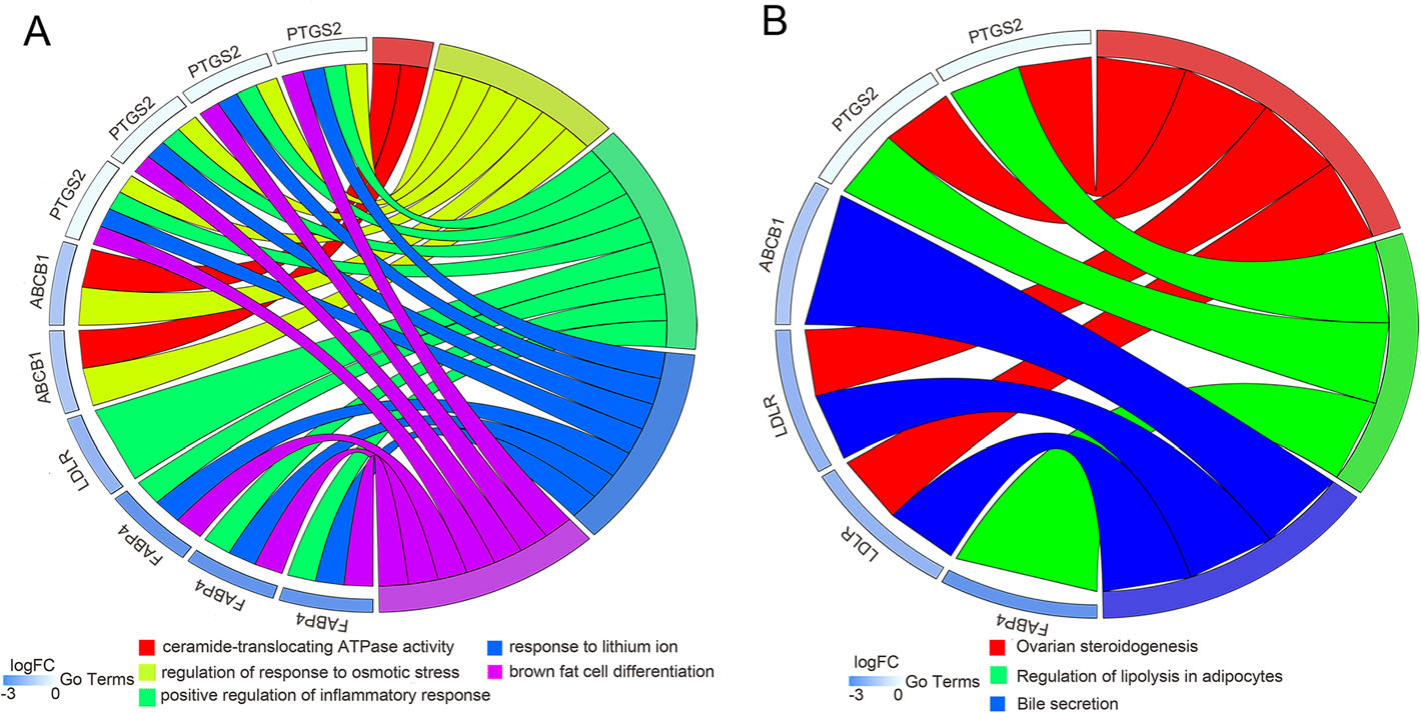
GO (**a**) and KEGG pathway (**b**) analyses of key targets

To further explore the multiple mechanisms of JOEI in NSCLC at the system level, we performed a GO enrichment analysis of five targets in the compound-NSCLC target network and identified five enriched GO terms (FDR < 0.01 and *P* < 0.01, as shown in Fig. 7a). Regarding biological processes, the potential targets were enriched in regulation of the response to osmotic stress (GO:0047484), positive regulation of the inflammatory response (GO:0050729), response to lithium ion (GO:0010226) and brown fat cell differentiation (GO:0050873). The analysis of molecular functions revealed that ceramide-translocating ATPase activity (GO:0099038) was particularly enriched.

**Fig. 8:**
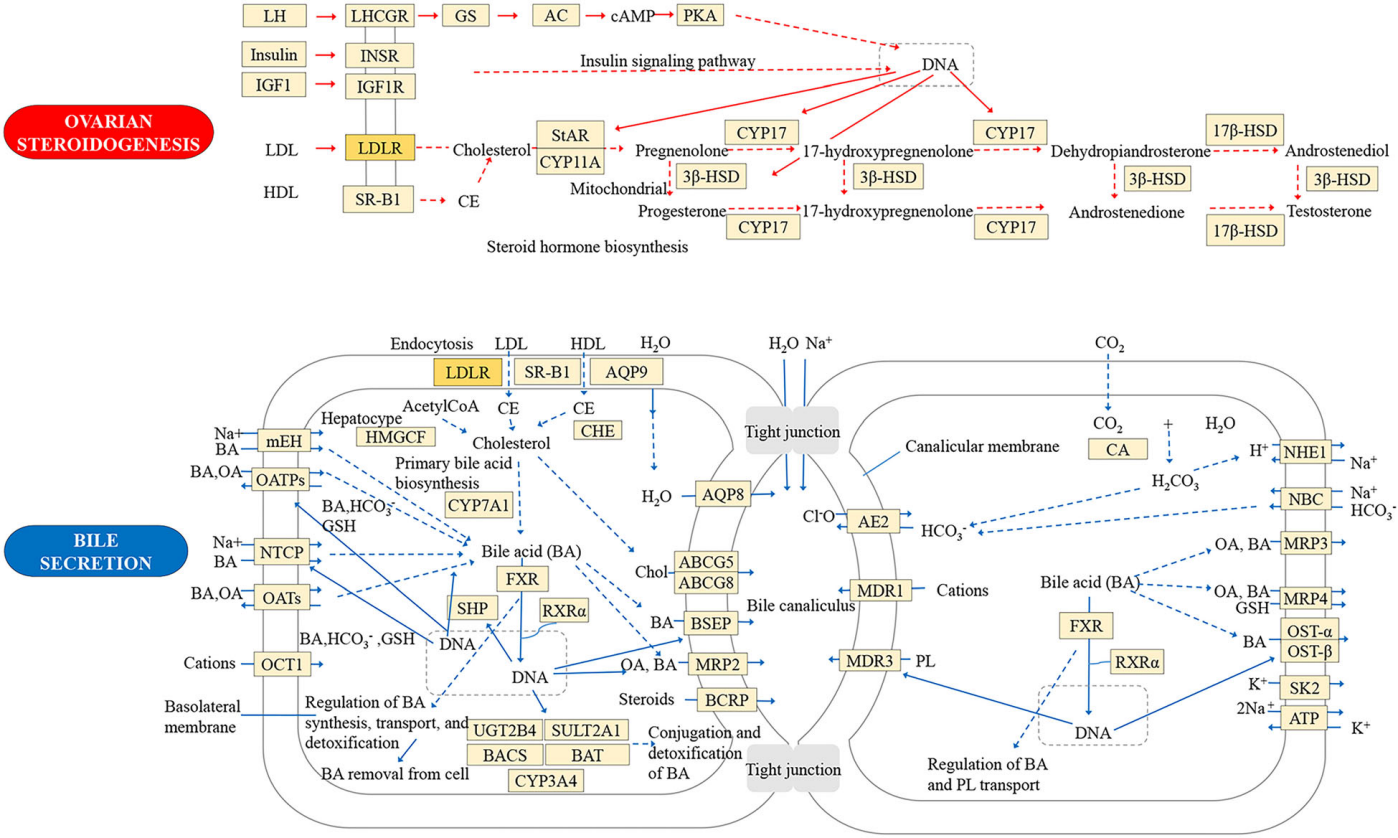
Graph of the interaction pathway

**Fig. 9:**
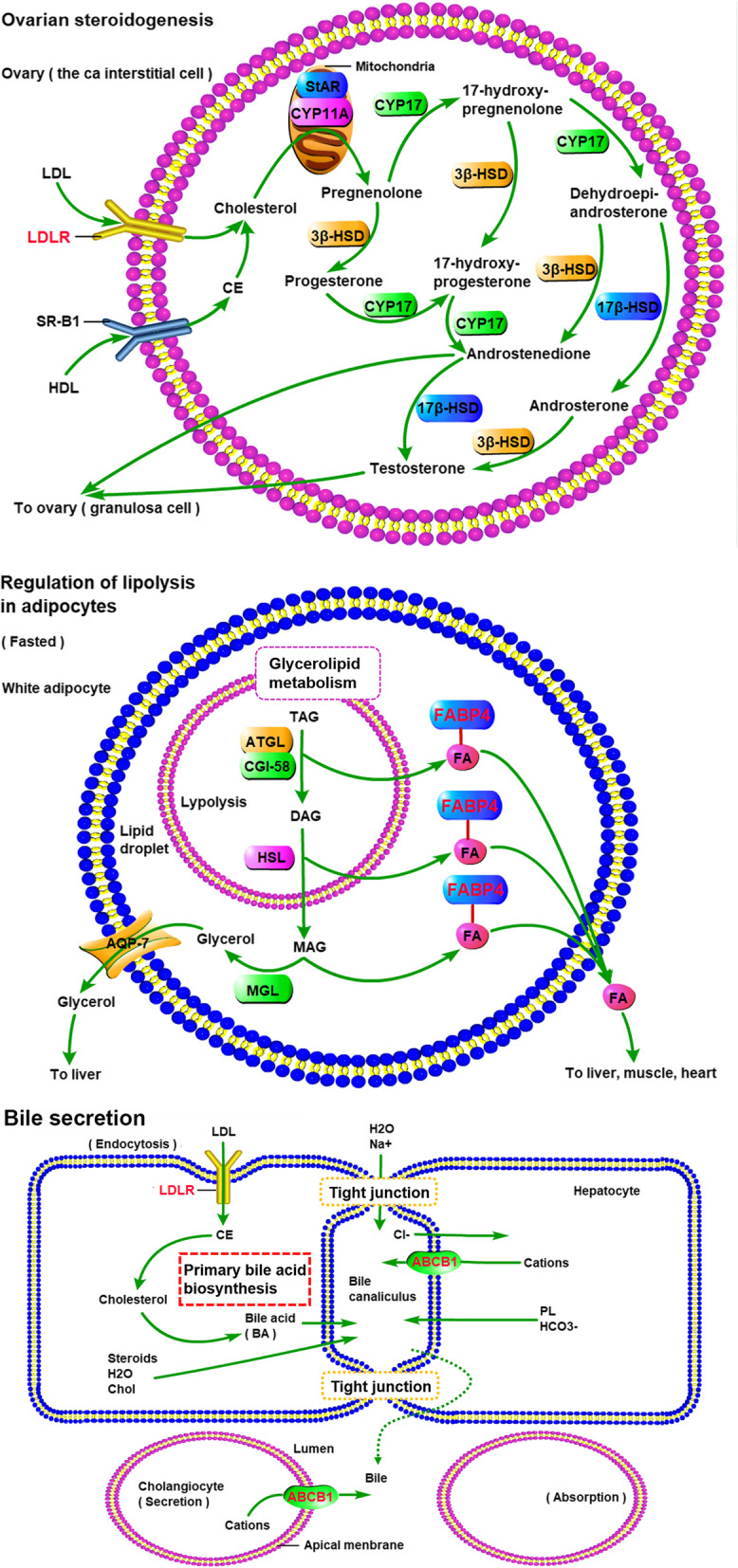
Signaling pathways

**Fig. 10:**
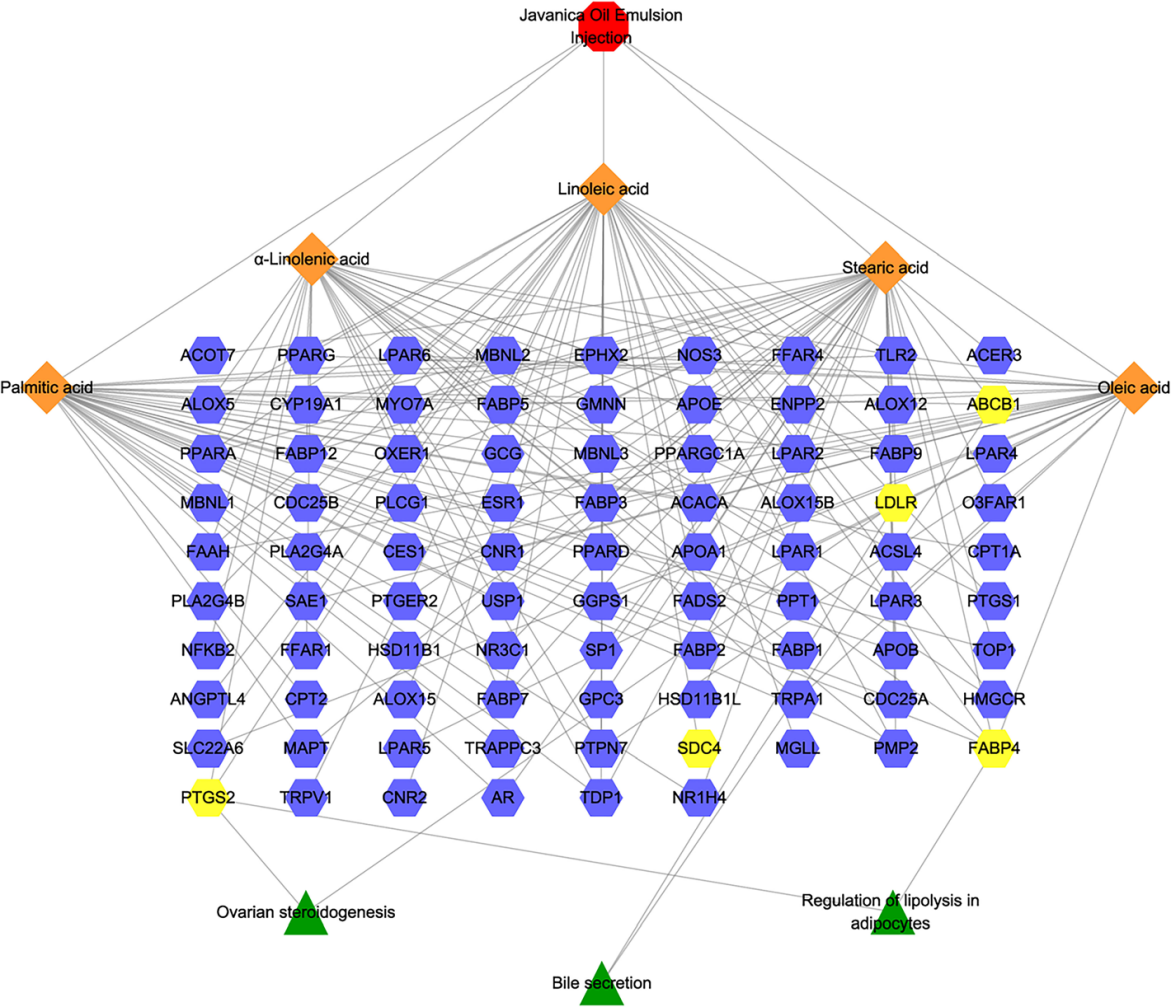
Compound-target-pathway network. Note: The red octagons represent JOEI; the five orange diamonds represent the chemical components of JOEI; the 97 purple hexagons represent the prediction targets; the common targets of JOEI and NSCLC are marked with yellow; and three green triangles represent the critical paths of the targets

To elucidate the crucial pathways among the five potential targets in NSCLC treatment, we identified three pathways based on the criteria FDR < 0.01 and *P* < 0.01 (as shown in Figs. 7b, 8 and 9): ovarian steroidogenesis (KEGG:04913), regulation of lipolysis in adipocytes (KEGG:04923) and bile secretion (KEGG:04976). Figure 10 shows the interactions between chemical components in JOEI and predictive targets and pathways of JOEI against NSCLC.

### Cox and survival analyses

**Fig. 11:**
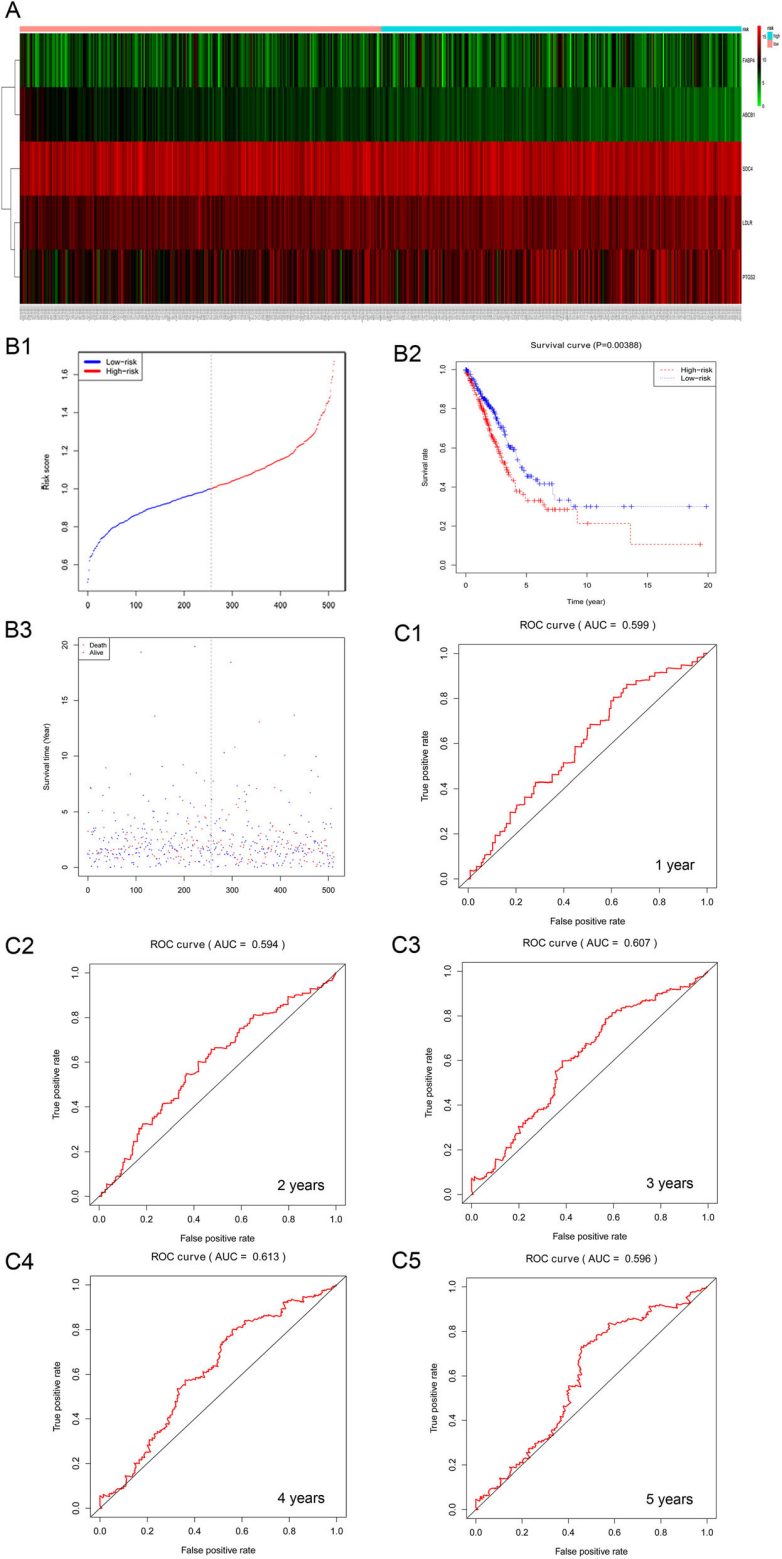
Heatmap of the five targets in patients in the low- and high-risk groups and prognostic validation of the core targets in the NSCLC cohort

The crucial genes were identified from the NSCLC cohort in TCGA. In both the high-risk group and the low-risk group, FABP4 and ABCB1 were weakly expressed, and SDC4, LDLR and PTGS2 were highly expressed (Fig. 11). The survival analysis showed that the survival rate of the high-risk group was fairly lower than that of the low-risk group (*p* = 0.00388). The AUC obtained from the survival analysis demonstrated that the crucial gene model exhibited the best predictive capacity over 4 years (AUC = 0.613) (Fig. 11).

### Molecular docking simulation

**Fig. 12:**
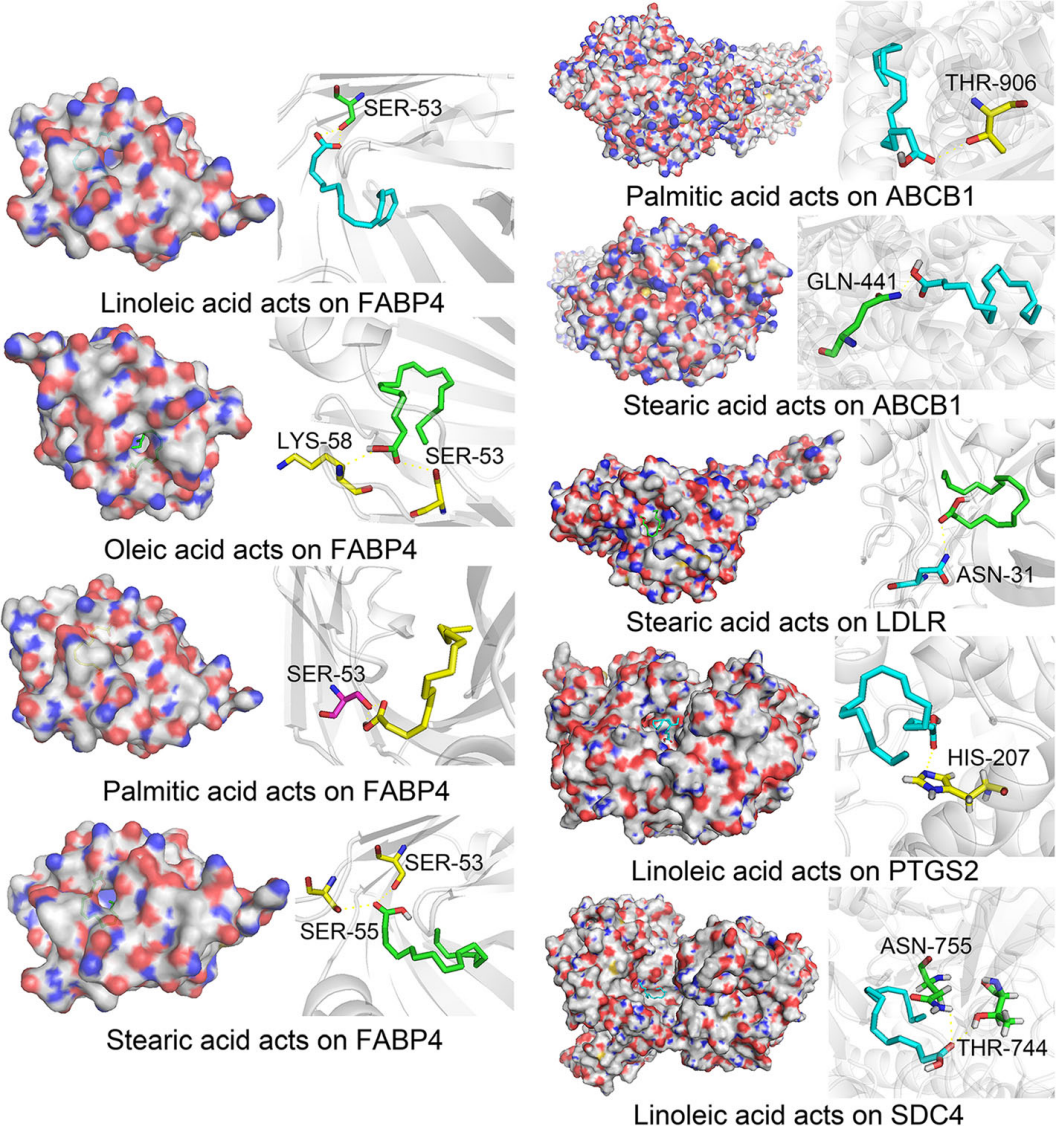
Detailed target-compound interactions obtained from the docking simulation

The mechanism of JOEI in the treatment of NSCLC was elucidated by investigating the interactions between compounds and targets. Therefore, the interactions between four compounds of JOEI (linoleic acid, oleic acid, palmitic acid, and stearic acid) and five targets (FABP4, ABCB1, LDLR, PTGS2 and SDC4) were investigated through molecular docking simulations. The 3D crystal structures of the five targets were derived from the PDB database based on their respective PDB codes. The results showed that four compounds exhibited relatively high potential for binding to the active sites of the five targets (Table 3). As shown in Fig. 12, hydrogen bond interactions were found between four compounds of JOEI and five targets.

**Table 3:** Docking information of five targets with their corresponding compounds

Target	PDB code	Ligand	Binding affinity (kcal/mol)
FABP4	5Y0F	Linoleic acid	−6.3
FABP4	5Y0F	Oleic acid	−5.8
FABP4	5Y0F	Palmitic acid	−5.9
FABP4	5Y0F	Stearic acid	−5.9
ABCB1	6C0V	Palmitic acid	−4.8
ABCB1	6C0V	Stearic acid	−4.3
LDLR	5OYL	Stearic acid	−3.1
PTGS2	5KIR	Linoleic acid	−4.2
SDC4	6EJE	Linoleic acid	−4.9

## Discussion

Based on histological characteristics, lung cancer can be divided into small cell lung cancer and NSCLC, and NSCLC accounts for 85% of all lung cancer cases [4, 59–61]. Despite great advances in the treatment of lung cancer, the OS rate of patients with NSCLC remains low [62]. As one of the crucial options for comprehensive cancer treatment, TCM has long been used to comprehensively treat NSCLC due to particular advantages, such as improving survival benefits, inhibiting tumor growth, and relieving postoperative symptoms and complications [63].

Explaining the mechanisms through which TCMs, as a multi-component, multi-target, and multi-pathway therapy, achieves its special therapeutic effect on the biological network of body systems is difficult [64, 65]. Favorably, network pharmacology presents a new perspective for cooperating with a new realization of the mechanisms of drugs and has become an dynamic method that incorporating systems biology, bioinformatics, and polypharmacology [22, 23, 66]. This approach updates the “one target, one drug” model to the “multi-component and multi-target” model, better elucidates the complex interactions among genes, proteins and metabolites during the drug treatment of diseases from a network perspective and provides evidence at the molecular biology level [67–69].

In this study, we implemented network pharmacology to identify bioactive components and targets of JOEI with the aim of identifying common targets in NSCLC and understanding and evaluating the underlying mechanism of JOEI in the treatment of NSCLC through expression level, correlation, enrichment, Cox, survival and molecular docking analyses.

According to the compound-NSCLC target network, five nodes are likely to be the core targets in NSCLC treatment. A previous study demonstrated that the FABP4 levels ae higher in NSCLC tissues than in normal tissues and that these high FABP4 levels have an unfavorable impact on the OS of NSCLC patients. Thus, the detection of FABP4 is helpful for predicting the prognosis of patients with NSCLC [70]. ABCB1 reportedly plays an important role in overcoming ABCB1-mediated docetaxel resistance in lung cancer [71]. Another study observed that ABCB1 is highly expressed in patients with stage I lung adenocarcinoma and that the expression of this protein is associated with poor survival, which indicates that ABCB1 expression is useful for predicting the prognosis of patients with lung adenocarcinoma [72]. Yang et al. identified ABCB1 as a vital downstream target of the chromosomal helicase/ATPase DNA-binding protein 1-like gene in NSCLC cells. The knockout of ABCB1 and ectopic expression of the chromosomal helicase/ATPase DNA-binding protein 1-like gene enhanced the effect of cisplatin on NSCLC cell apoptosis [73]. Regarding LDLR, Yang et al. demonstrated that T lymphocytes (LMPs) exert antiangiogenic and proapoptotic effects that lead to inhibition of lung carcinoma by decreasing the vascular endothelial growth factor levels, and the knockdown of LDLR reduces the uptake of LMPs by Lewis lung carcinoma cells and attenuates the inhibitory effects of LMPs on cell growth and vascular endothelial growth factor expression. These results show that LMPs portray a new treatment protocol for treating lung carcinomas and indicate that LDLR plays an important role [74]. With respect to PTGS2/COX-2, Jiang et al. indicated that PTGS2/COX-2 might promote cisplatin resistance in NSCLC by favoring epithelial-mesenchymal transition through activation of the AKT signaling pathway [75]. Previous studies have shown that the inhibition of PTGS2 might play a beneficial role in the treatment of NSCLC, such as improving the overall response rate of advanced NSCLC and suppressing the metastasis of lung cancer cells [76–79]. Some data suggest that PTGS2 is likely a potential prognostic marker for unresectable NSCLC [80]. Concerning SCD4, high Toll-like receptor 7 expression is associated with the overexpression of SDC4 in patients with adenocarcinoma, which suggests that its expression is related to metastasis. SDC4 also plays a role in the occurrence and metastasis of renal cell carcinoma [49, 81, 82]. Repressor of silencing 1 (ROS1) protein-tyrosine kinase fusion proteins are expressed in 1–2% of NSCLC patients [83]. For NSCLC patients with tumors expressing the ROS1 fusion gene, ROS1 inhibition might be an effective treatment protocol, and SDC4 plays a vital role as a common ROS1 fusion partner [84, 85].

The compound-putative target network included linoleic acid, α-linolenic acid, palmitic acid, stearic acid and oleic acid. We found that linoleic acid induces the expression of PTGS2 in retinal pigment epithelial cells at the mRNA and protein levels in a time- and dose-dependent manner [86]. PTGS2 expression is a powerful predictor of NSCLC [87]. In NSCLC, an allele of chromosome 3p is often lost, which confirms the existence of cancer suppressor genes in this chromosomal region. We found that a Fus1 peptide inhibits ABL tyrosine kinase in vitro. The repressive Fus1 sequence stems from a deleted region of the mutant Fus1 gene detected in lung cancer cell lines, and notably, a stearic acid-modified form of this peptide is required for inhibition [88]. Epidermal growth factor receptor (EGFR) tyrosine kinase inhibitors are effective in the treatment of NSCLC patients with EGF mutations, but resistance is inevitable. The cytotoxic effect of both gefitinib and osimertinib in EGFR-activated mutant cell lines can be inhibited by oleic acid [89]. The FABP4 inhibitor inhibited cell growth induced by oleic acid, leptin, vascular endothelial growth factor, and DHA (*P* < 0.05). The levels of FABP4 protein in these cells are increased by oleic acid vascular endothelial cells and leptin [90]. An increase in the miR-146b-5p level is related to decreased FABP4 expression, glucose metabolism and FABP4 mobilization. In partial agreement with these findings, palmitic acid lead to decreased miR-146a levels in vitro; thus, FABP4 is associated with palmitic acid [91], which is consistent with the results of our study.

In this study, we performed enrichment analyses to clarify the multiple mechanisms of JOEI in the treatment of NSCLC at the system level. We found that the pathways directly related to lung cancer were ovarian steroidogenesis, regulation of lipolysis in adipocytes and bile secretion. The production of ovarian steroidogenesis is vital for the normal function of the uterus, the establishment and maintenance of pregnancy and the development of the mammary gland; it also demands cooperative interactions between the theca and granulosa cells within the follicle [92]. Morphological examinations play important roles in the assessment of ovarian steroidogenesis, particularly in patients with ovarian tumors associated with abnormal sexual steroids [93]. Increased lipogenesis is one of the most important metabolic characteristics of cancer cells. Recent findings have revealed that breast and liposarcoma cancers have both de novo fatty acid synthesis pathways and lipoprotein lipase-mediated extracellular lipolysis. Nonetheless, recent studies have shown that the proliferation and survival of cancer cells are affected by fatty acids, and some cancer cells/tissues can obtain fatty acids through adipogenesis and lipolysis [94–96]. Bile normally functions to emulsify and facilitate the intestinal absorption of dietary fats, protect the organism from enteric infections by excreting immune globulin A and inflammatory cytokines, and stimulate the innate immune system in the intestine. Bile secretion plays a vital role in the health of an organism, and therefore, disrupted bile secretion can eventually result in liver failure or death [97].

## Conclusion

In conclusion, the current study explored and predicted the molecular mechanism of JOEI against NSCLC using a network pharmacology and bioinformatics approach. We hope that our study will lay a good foundation for further experimental studies and contribute to the application of network pharmacology for exploring the potential mechanisms of complex diseases. However, because this study was based on data analysis, further experimental data from in vitro and in vivo experiments are needed to verify the findings and optimize the method.

## Supplementary information

**Additional file 1: Supplementary 1.** Proteins related to NSCLC (*n* = 406).

**Additional file 2: Supplementary 2.** List of GO enrichment results of potential targets of JOEI associated with NSCLC.

**Additional file 3: Supplementary 3.** List of KEGG enrichment results of potential targets of JOEI associated with NSCLC.

**Additional file 4: Supplementary 4.** RSs of candidate mRNAs for each NSCLC patient.

## Data Availability

The datasets used and/or analyzed during the current study are available from the corresponding author upon reasonable request.

## Abbreviations

ADT: AutoDockTools
AUC: Area under the curve
DEGs: Differentially expressed genes
GEO: The gene expression omnibus
GEPIA: Gene expression profiling interactive analysis
GTEx: Genotype-tissue expression
GO: Gene ontology
JOEI: Javanica oil emulsion injection
KEGG: Kyoto encyclopedia of genes and genomes
NSCLC: Non-small cell lung cancer
OS: Overall survival
TCM: Traditional Chinese medicine
TTD: Therapeutic target database
RCSB: Research Collaboratory for Structural Bioinformatics
RMSD: Root mean square deviation
ROC: Receiver operating characteristic
RS: Risk score
